# An aMMP-8 Point-of-Care and Questionnaire Based Real-Time Diagnostic Toolkit for Medical Practitioners

**DOI:** 10.3390/diagnostics11040711

**Published:** 2021-04-15

**Authors:** Ismo T. Räisänen, Hanna Lähteenmäki, Shipra Gupta, Andreas Grigoriadis, Vaibhav Sahni, Juho Suojanen, Hanna Seppänen, Taina Tervahartiala, Dimitra Sakellari, Timo Sorsa

**Affiliations:** 1 Department of Oral and Maxillofacial Diseases, University of Helsinki and Helsinki University Hospital, 00014 Helsinki, Finland; hanna.maa.lahteenmaki@gmail.com (H.L.); taina.tervahartiala@helsinki.fi (T.T.); timo.sorsa@helsinki.fi (T.S.); 2 Unit of Periodontology, Oral Health Sciences Centre, Post Graduate Institute of Medical Education & Research (PGIMER), Chandigarh 160012, India; shipra1472@gmail.com; 3 Department of Preventive Dentistry, Periodontology and Implant Biology, Dental School, Aristotle University of Thessaloniki, 54124 Thessaloniki, Greece; andreasgrigor@gmail.com; 4 424 General Military Training Hospital, 54124 Thessaloniki, Greece; dimisak@med.auth.gr; 5 Department of Periodontics, Dr. Harvansh Singh Judge Institute of Dental Sciences & Hospital, Panjab University, Chandigarh 160014, India; vaibhav.sahni@rcsed.net; 6 Cleft Palate and Craniofacial Center, Department of Plastic Surgery, Helsinki University Hospital, 00029 Helsinki, Finland; juho.suojanen@helsinki.fi; 7 Department of Oral and Maxillo-Facial Surgery, Päijät-Häme Joint Authority for Health and Wellbeing, 15850 Lahti, Finland; 8 Department of Surgery, University of Helsinki and Helsinki University Hospital, 00029 Helsinki, Finland; hanna.seppanen@hus.fi; 9 Translational Cancer Medicine Research Program, University of Helsinki, 00014 Helsinki, Finland; 10 Division of Periodontology, Department of Dental Medicine, Karolinska Institutet, 14104 Huddinge, Sweden

**Keywords:** periodontitis, diagnostics, point-of-care systems, biomarkers, matrix metalloproteinase 8, preventive medicine, oral health

## Abstract

The aim of this cross-sectional study is to propose an efficient strategy based on biomarkers adjunct with an interview/questionnaire covering risk factors for periodontitis for the identification of undiagnosed periodontitis by medical professionals. Active matrix metalloproteinase (aMMP)-8 levels in mouthrinse were analyzed by a point-of-care (PoC)/chairside lateral-flow immunotest, and salivary total MMP-8, total MMP-9 and calprotectin levels were analyzed by enzyme-linked immunosorbent assays (ELISAs) and active MMP-9 by gelatin zymography for 149 Greek patients. Patients underwent a full-mouth oral health examination for diagnosis according to the 2018 classification system of periodontal diseases. In addition, patient characteristics (risk factors: age, gender, education level, smoking and body mass index) were recorded. Receiver operating curve (ROC) analysis indicated better diagnostic precision to identify undiagnosed periodontitis for oral fluid biomarkers in adjunct with an interview/questionnaire compared with a plain questionnaire (i.e., risk factors): aMMP-8 AUC (95% confidence interval) = 0.834 (0.761−0.906), total MMP-8 = 0.800 (0.722–0.878), active MMP-9 = 0.787 (0.704–0.870), total MMP-9 = 0.773 (0.687−0.858) and calprotectin = 0.773 (0.687–0.858) vs. questionnaire = 0.764 (0.676–0.851). The findings of this study suggest that oral fluid biomarker analysis, such as a rapid aMMP-8 PoC immunotest, could be used as an adjunct to an interview/questionnaire to improve the precision of timely identification of asymptomatic, undiagnosed periodontitis patients by medical professionals. This strategy appears to be viable for referring patients to a dentist for diagnosis and treatment need assessment.

## 1. Introduction

The link between periodontitis and several systemic diseases (cancers, diabetes, cardiovascular diseases (CVDs), Alzheimer’s disease, etc.) and induced low-burden systemic inflammation makes periodontitis an important disease not only for dentists but also for medical professionals [1,2,3,4,5,6,7]. However, it is unfeasible for a medical professional to detect periodontitis due to a lack of both the equipment required for intraoral examination, as well as professional knowledge regarding oral diseases and their diagnosis [8,9]. Most are yet to be sensitized regarding the oral-systemic links and the importance of oral health maintenance. In rare cases, some of the most knowledgeable professionals may check for oral hygiene or breath odor. As a result, the most obvious cases of periodontitis with bleeding gums and stark halitosis may get identified. However, as these symptoms are typically associated with more severe stages of periodontitis, those who are asymptomatic will generally go undiagnosed.

Currently, the concept of interviewing the patient has been put forward as a way to identify patients at risk of periodontitis [8,9]. The interview covers risk factors for periodontitis such as smoking and bleeding gums that could aid in identification of patients at risk of periodontitis [8,9]. Furthermore, a large number of studies have investigated oral fluid biomarkers for diagnosis of periodontitis [10,11]. These include, for example, several proteolytic enzymes involved in degradation of connective tissue such as active matrix metalloproteinase-8 (aMMP-8) and MMP-9; and their activators and inhibitors such as myeloperoxidase (MPO), calprotectin, and tissue inhibitor of metalloproteinases 1 (TIMP-1); as well as bioactive proinflammatory and immunoregulatory cytokines such as interleukin-1β (IL-1β), interleukin-6 (IL-6), and transforming growth factor β1; and cell surface receptors part of the signaling pathways such as triggering receptor expressed on myeloid cells-1 (TREM-1) and soluble urokinase-type plasminogen activator receptor (suPAR) [10,11,12,13,14,15,16,17]. They form a complex network that orchestrates and regulates the inflammatory immune response and its magnitude in periodontal tissues [10,11,12,13,14,15,16,17].

Previous studies have established the role of aMMP-8 in the progression and disease activity of periodontitis [18,19,20,21,22,23,24,25]. They have also shown that aMMP-8 levels correlate well with clinical periodontal indices and parameters [18,19,20,21,22,23,24,25]. This fact could prove to be important in the timely identification of asymptomatic, undiagnosed periodontitis patients. A pathological increase in aMMP-8 concentration in oral fluids is a strong risk factor and reflective of active periodontal disease, which can be measured at the point-of-care/chairside in 5–10 min without professional dental expertise or equipment [2,3,22,23,24,25,26]. Elevated aMMP-8 levels are a covert sign of periodontitis, the activation and progression of which cannot be identified by interviews alone.

The aim of this study was to evaluate the potential benefits of using biomarkers (active MMP-8, total MMP-8, active MMP-9, total MMP-9 and calprotectin) in adjunct with such (potential) risk factors for periodontitis that are easily obtainable by any medical practitioner—namely, gender, age, education level, smoking and body mass index (BMI). Specifically, our hypothesis was that aMMP-8, which currently has available a rapid point-of-care (PoC)/chairside technology, could help to improve the accuracy of identification of undiagnosed periodontitis by medical professionals when combined with interviews and/or questionnaires covering these risk factors.

## 2. Materials and Methods

This cross-sectional study with 150 Greek participants (age 25–78 years) was conducted in the Department of Periodontology, Dental School, Aristotle University, Thessaloniki, Greece, and the Periodontal Department of 424 General Army Hospital, Thessaloniki, Greece. Participants signed an informed consent form and the study was conducted according to the protocol outlined by the Research Committee, Aristotle University of Thessaloniki, Greece, and approved by the Ethical Committee of the School of Dentistry (protocol number #64, 06/12/2018) and in accordance with the Declaration of Helsinki. Patients over 18 years old fulfilling the Centers for Disease Control and Prevention (CDC, USA) criteria and who were at risk for prediabetes were included in the study [2,3,27]. Patients with any underlying diseases or using antibiotic or anti-inflammatory medications in the last 3 months before the examination were excluded from this study.

Oral health status was recorded for all patients by one calibrated examiner (A.G.), as described previously [2,3]. Periodontitis was classified by the 2018 classification system of periodontal diseases [28,29]. Patients were dichotomized: Stages II–III (moderate–severe) of periodontitis (“yes”) (n = 104) vs. patients with no periodontitis or Stage I (initial) periodontitis (“no”) (n = 46). Before the conduction of oral health examination, an aMMP-8 PoC/chairside lateral-flow immunotest (PerioSafe® test, Dentognostics GmbH, Jena, Germany combined by the digital reader ORALyzer®, Dentognostics GmbH, Jena, Germany) was used to measure aMMP-8 levels in mouthrinse, while enzyme-linked immunosorbent assay (ELISA) kits (Quantikine, R&D Systems, Minneapolis, MN, USA) were used for salivary total MMP-8 (detection limit of 0.013 ng/mL), total MMP-9 (detection limit of 0.156 ng/mL) and calprotectin levels (detection limit of 0.215 ng/mL), and zymographic technique for active MMP-9, as described previously [3,25,30,31]. Finally, patient data were recorded in terms of risk factors for periodontitis (questionnaire): gender, age, education level, smoking and BMI [3].

To evaluate diagnostic ability of the aMMP-8 PoC test, as well as total MMP-8 and calprotectin ELISAs for periodontitis in adjunct with an interview/questionnaire (gender, age, education level, smoking and BMI), multivariable logistic regression models were calculated. The four models were compared in the receiver operating curve (ROC) analysis. To assess the quality of classification by each model, optimal cut-offs based on the Youden index were defined from the ROC curves and used to calculate diagnostic sensitivity (Se), specificity (Sp), the percentage of false negatives (FNs) and false positives (FPs), test accuracy (Acc), Matthews correlation coefficient (MCC) and F1 score. There was a missing value for one patient related to the age and BMI, which left a total sample size of 149 patients. Statistical significance was set at *p* values ≤ 0.05. Statistical analyses were performed using SPSS software, version 27 (IBM Corp., Armonk, NY, USA).

## 3. Results

**Figure 1:**
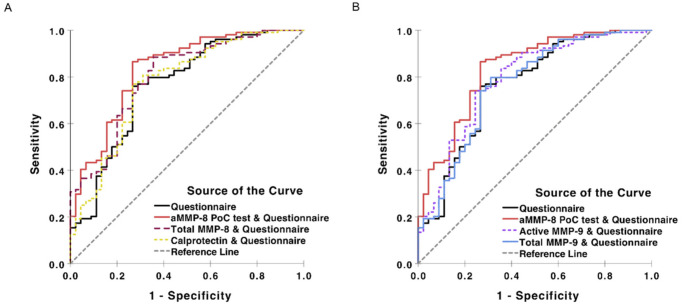
Receiver operating curve (ROC) analysis regarding using questionnaire and biomarkers (**aMMP-8**, total **MMP-8**, active **MMP-9**, total **MMP-9** and calprotectin) to identify undiagnosed periodontitis (Stages II–III) (n = 149 Greek patients, as described previously [2,3,24]). (**A**) A plain questionnaire vs. a questionnaire adjunct with **aMMP-8**, total **MMP-8**, or calprotectin, and (**B**) a plain questionnaire vs. a questionnaire adjunct with **aMMP-8**, active **MMP-9**, or total **MMP-9**. Questionnaire includes patient’s gender, age, education level, smoking and body mass index (BMI).

ROC analysis of logistic regression models (Figure 1 and Table 1) indicated that biomarkers have good potential to increase the accuracy that a plain questionnaire offers for identification of undiagnosed periodontitis patients: aMMP-8 AUC (95% CI) = 0.834 (0.761–0.906), total MMP-8 = 0.800 (0.722–0.878), active MMP-9 = 0.787 (0.704–0.870), total MMP-9 = 0.767 (0.680–0.855) and calprotectin = 0.773 (0.687–0.858) adjunct with the questionnaire vs. plain questionnaire = 0.764 (0.676–0.851). aMMP-8 levels indicating active collagenolysis and periodontal degeneration had the best precision when adjunct with the questionnaire compared with total MMP-8, active MMP-9, total MMP-9 and calprotectin and the plain questionnaire to improve the identification of the undiagnosed periodontitis cases that need to be referred to a dentist (Figure 1 and Table 1).

## 4. Discussion

Our results show that oral fluid biomarker analysis in adjunct with an interview/questionnaire, covering risk factors for periodontitis, could be a feasible tool for the timely identification of asymptomatic, undiagnosed periodontitis patients by medical professionals. Oral fluid biomarker analysis in adjunct with an interview/questionnaire was able to increase the accuracy offered by a plain questionnaire. It should be noted that, unlike questionnaires, biomarkers such as aMMP-8 could provide medical practitioners real-time and online information about the current disease activity. Thus, combination of questionnaires and emerging rapid point-of-care biomarker technologies could further benefit medical practitioners in the identification of at-risk patients. This is especially important when regarding patients with certain systemic diseases. The impact of periodontitis on diabetes and CVD is well underscored in the latest consensus guidelines of these diseases [4,6]. Periodontitis can increase the risk of low-burden systemic inflammation, affecting, for example, the maintenance of diabetes or complications of atheromatous cardiovascular disease [2,3,4,6,32].

In the same vein, our findings suggest that using an interview/questionnaire covering risk factors for periodontitis and an aMMP-8 PoC/chairside test together had the best potential to identify patients with undiagnosed periodontitis. Furthermore, active MMP-8 levels had better precision than total MMP-8 levels, while active MMP-9, total MMP-9 and calprotectin showed minimal incremental values as adjuncts to the questionnaire. In this regard, a prospective study by Lee et al. was one of the first to demonstrate longitudinally that it is the active form of MMP-8 (aMMP-8) that plays the major role in pathological destruction of periodontal connective tissues [18,19,20,21]. However, studies with total MMP-8 levels (latent + active) have given controversial results, which suggests that total MMP-8 levels may not be as accurate/reliable measure as active MMP-8 in periodontal disease diagnostics [18,19,20,22,23,24,33,34,35,36,37].

The strategy of using oral fluid point-of-care testing of periodontitis in adjunct with interviews/questionnaires covering risk factors for periodontitis has several potential benefits for medical practitioners. It may increase the precision of identification of at-risk patients, while information regarding disease activity could further help to assess and prioritize patients’ needs and urgency to see a dentist for periodontal evaluation. This could be particularly useful among patients with diabetes and CVD, who are at greater risk for complications with periodontitis [4,6]. The aMMP-8 PoC test is rapid, easy to use, and does not require dental expertise [2,3,22,23,24,25,26]. Thus, it is well suited for medical professionals in addition to being of utility to oral healthcare providers [20]. This kind of point-of-care testing requires only the collection of an oral fluid sample, which is a noninvasive and nonbacteraemic method that requires no antibiotic prophylaxis and is painless for patients [2,3,22,23,24,25,26,37]. To the best of our knowledge, regarding the biomarkers in this study, in addition to aMMP-8, there exists oral fluid point-of-care/chairside tests only for MMP-9 and calprotectin [38,39].

A possible limitation in this study is that patients with underlying diseases or using antibiotic or anti-inflammatory medications were excluded. Recently, Rautava et al. found that Crohn’s disease, which is an autoimmune disease, and immunomodulatory/autoinflammatory medications related to that disease may decrease the strength of the association between periodontitis and aMMP-8 levels in oral fluids compared with healthy controls [40]. On the other hand, a pilot study by Keskin et al. found an association between elevated aMMP-8 levels and progression of periodontitis in head and neck cancer patients treated by radiotherapy [26]. Nevertheless, further research in different populations is still needed to extend our knowledge in this area.

In conclusion, biomarkers and particularly aMMP-8 PoC testing in adjunct with interviewing/questionnaires seem to offer great potential to open up new possibilities for medical professionals to identify the risk of “hidden” periodontitis in their patients and refer them to a dentist in order to institute necessary care. This would strengthen the identification of patients at risk of periodontitis to better ensure its prevention and treatment.

## Figures and Tables

**Table 1:** Diagnostic potential of a questionnaire and biomarkers (aMMP-8, total MMP-8, active MMP-9, total MMP-9 and calprotectin) to identify periodontitis (Stages II–III). The Youden index was used defining the optimal cut-offs for each logistic regression model from the ROC curves.

Model	AUC (95% CI)	*p* Value	Cut-off Point	Se (%)	Sp (%)	FN (%)	FP (%)	Acc (%)	MCC	F1 Score
Questionnaire	0.764 (0.676–0.851)	<0.001	0.656	76.0	73.3	43.1	13.2	75.2	0.464	0.810
aMMP-8 PoC test and Questionnaire	0.834 (0.761–0.906)	<0.001	0.574	86.5	73.3	29.8	11.8	82.6	0.592	0.874
Total MMP-8 and Questionnaire	0.800 (0.722–0.878)	<0.001	0.570	88.5	64.4	29.3	14.8	81.2	0.544	0.868
Active MMP-9 and Questionnaire	0.787 (0.704–0.870)	<0.001	0.692	74.0	75.6	44.3	12.5	74.5	0.463	0.802
Total MMP-9 and Questionnaire	0.767 (0.680–0.855)	<0.001	0.625	79.8	68.9	40.4	14.4	76.5	0.469	0.826
Calprotectin and Questionnaire	0.773 (0.687–0.858)	<0.001	0.666	76.9	73.3	42.1	13.0	75.8	0.475	0.816

Questionnaire: gender, age, education level, smoking, body mass index (BMI). **AUC**: Area Under the ROC Curve; **CI**: confidence interval; **Se**: sensitivity; **Sp**: specificity; **FN**: false negatives; **FP**: false positives; **Acc**: accuracy; **MCC**: Matthews correlation coefficient; POC: point of care; **F1 score**: the harmonic mean of the precision and recall. ***p*** values calculated by the Mann-Whitney U test.

## Data Availability

The data that support the findings of this study are available on reasonable request from the corresponding author. The data are not publicly available due to privacy and ethical restrictions.

## Sample Availability

Samples of the compounds have been all consumed and are not anymore available from the authors.
